# The Physician’s Visiting List, Diary, and Book of Engagements, for 1859

**Published:** 1858-11

**Authors:** 


ART. VIII.—*The Physician's Visiting List, Diary, and Book of Engagements, for* 1859. Lindsay and Blakiston: Philadelphia.

WE have received from the publishers this convenient little pocket-book, and would recommend it to every physician, although it is unnecessary, as it is already so well known to the profession. In addition to the usual contents, the "Marshall Hall ready method in Asphyxia" finds a place in this edition.

